# Prevalence of coinfections in women living with human immunodeficiency virus in Northeast Brazil

**DOI:** 10.1590/0037-8682-0282-2019

**Published:** 2019-12-20

**Authors:** Brenda Evelin Barreto da Silva, Victor Santana Santos, Ingrid Emmily Reis Santos, Marcus Vinicius de Aragão Batista, Leila Luiza Conceição Gonçalves, Lígia Mara Dolce de Lemos

**Affiliations:** 1 Universidade Federal de Sergipe, Programa de Pós-Graduação em Enfermagem, São Cristóvão, SE, Brasil.; 2 Universidade Federal de Alagoas, Núcleo de Epidemiologia e Saúde Pública, Arapiraca, AL, Brasil.; 3 Universidade Federal de Sergipe, Departamento de Enfermagem, Aracaju, SE, Brasil.; 4 Universidade Federal de Sergipe, Departamento de Biologia, São Cristóvão, SE, Brasil.

**Keywords:** Coinfection, HIV, Women

## Abstract

**INTRODUCTION:**

Despite the success of antiretrovirals, human immunodeficiency virus (HIV) coinfections continue to cause mortality. We investigated the prevalence of coinfections in women with HIV/acquired immunodeficiency syndrome in Sergipe, Brazil.

**METHODS:**

We conducted a cross-sectional study. The coinfections investigated were syphilis, hepatitis B and C, toxoplasmosis, rubella, tuberculosis, and cytomegalovirus.

**RESULTS:**

Among the 435 women, 85 (19.5%) had coinfections. The most prevalent was HIV/syphilis, followed by tuberculosis, toxoplasmosis, hepatitis C, hepatitis B, and rubella. Additionally, 300 (96.2%) were seropositive for cytomegalovirus immunoglobulin G.

**CONCLUSIONS:**

Despite significant progress in the treatment for people with HIV, coinfections continued to affect this population.

Coinfections with human immunodeficiency virus (HIV) continue to be widely discussed worldwide^1^. With the introduction of antiretroviral therapy (ART), there has been a change in the natural pattern of development of HIV infection, which led to a reduction in morbidity and mortality. However, HIV coinfections are still observed, leading to hospital admissions and preventable death^2^.

In Brazil, although the prevalence of HIV is higher in men than that in women, women comprise an increasing proportion of the population affected by HIV, and approximately 4,000 women die from acquired immunodeficiency syndrome (AIDS)-related illnesses annually^3^. In this context, coinfections among HIV-infected women are concerning, specifically for women in reproductive age or who are pregnant. Furthermore, serious complications or death of the women and/or fetus can be caused by worsening of the HIV infection^4^.

In this study, we investigated the prevalence of coinfections in women living with HIV/AIDS attended at a reference outpatient clinic for individuals living with HIV/AIDS in Sergipe State, Northeast Brazil.

We conducted a cross-sectional study from August 2014 to November 2017 in women living with HIV/AIDS who attended the Reference Centre for Sexually Transmitted Diseases, HIV, and AIDS (CRIST/AIDS), the unique reference care center for individuals with HIV in Sergipe State, Northeast Brazil.

The sample size was calculated based on the number of HIV-positive women registered at the CRIST/AIDS in 2014 (800 women). The prevalence of the expected coinfections was 50%, confidence interval (CI) was 95%, and maximum error was 5%, which resulted in 260 women. However, at the end of the study, 435 HIV-positive women were included.

Data were collected through individual interviews with a structured form. For the viral load and CD4+ T-lymphocyte count, only the data obtained less than 1 year prior to the interview were included.

Patients diagnosed with HIV infection in the CRIST/AIDS underwent initial complementary examinations including tests for syphilis, viral hepatitis, toxoplasmosis, tuberculosis (TB), cytomegalovirus, and rubella. In this study, we opted to analyze these coinfections that were screened through routine examinations of the service itself. We retrieved information from all medical records from HIV diagnosis until the interview’s date. Additionally, the Brazilian Information System for Notifiable Diseases (SINAN) database of TB was used.

*Toxoplasma gondii* and *Rubella* virus immunoglobulin M (IgM) and immunoglobulin G (IgG) antibodies were recorded; however, only the IgG test results were considered for cytomegalovirus because IgM test had been requested less frequently in the clinic. Syphilis was screened using treponemal and non-treponemal tests. Positive hepatitis B and C diagnosis was established with the following serological markers: hepatitis B surface antigen (HBsAg) and hepatitis C antibody. Finally, to identify the occurrence of HIV-TB coinfection, record linkage was performed between the database of this study and the SINAN database with TB cases among women in Sergipe between 2001 and 2017.

The characteristics were analyzed using descriptive statistics. The prevalence of HIV coinfections was described as a simple proportion. Pearson’s chi-squared test and Fisher’s exact test were used to compare the association between coinfection and the time since HIV diagnosis. The significance level was set at 5%. For the association between predictor factors and the occurrence of coinfections, the prevalence ratio (PR) with a 95% CI was used. The data were analyzed using the Statistical Package for the Social Sciences version 20.0 (International Business Machines Corporation, Armonk, NY).

This study was approved by the Research Ethics Committee of the Federal University of Sergipe (CAAE No. 92514618.8.0000.5546) and following the Helsinki Declaration. All participants provided written informed consent. Parents or guardians provided written informed consent before enrolling their children in the study.

The age of the 435 HIV-seropositive women ranged from 13 to 76 years, with a median age of 38 years (interquartile range, 30-46 years); 38 (88.2%) women had less than 8 years of education, 280 were married (67.6%), and 338 (78.3%) had 1-2 minimum wage. Of the 435 women, 329 (75.6%) had been infected sexually and 191 (45.4%) had their first sexual intercourse when they were younger than 15 years old. Most of them were diagnosed with HIV infection more than 5 years (228/52.4%), 309 (77.1%) had a CD4+ T-lymphocyte count higher than 350 cells/μl, 309 (76.7%) had HIV viral load from zero to 999 copies/mL, and 414 (95.6%) reported the use of antiretrovirals (Table 1).

**TABLE 1:** Sociodemographic, economic, clinical, and risk behavior characteristics of women living with HIV^a^, Sergipe, Brazil, August 2014-November 2017.

Characteristics	N^c^	%
**Age group (years old) (n= 435)**		
13-25	63	14.5
26-49	298	68.5
≥50	74	17.0
**Years of education (n= 431)**		
≤8	380	88.2
>8	51	11.8
**Race (n= 396)**		
White	60	15.3
Black	120	30.5
Mixed	213	54.2
**Conjugal union (n= 414)**	280	67.6
**Occupation (n= 435)**		
Employed	133	30.6
Unemployed	77	17.7
Benefit salary^b^	96	22.0
Housewives/students	129	29.7
**Household income (n= 432)**		
No income	33	7.6
1-2 salaries	338	78.3
>2 salaries	61	14.1
**Sexual partner (n= 435)**		
Steady partner	274	63.0
Casual partner	28	6.4
Steady and casual partner	4	0.9
No partner	129	29.7
**Number of sexual partners in the last year (n= 401)**		
No partner	75	18.7
1 or 2	301	75.1
>2	25	6.2
**HIV exposure category (n= 435)**		
Sexual intercourse	329	75.6
Vertical transmission	7	1.6
Unknown	99	22.8
**Drug use (n= 433)**	78	18.0
**Sex for money (n= 430)**	45	10.5
**Condon use (n= 409)**	172	42.1
**First sexual intercourse ≤15 years (n= 421)**	191	45.4
**Number of pregnancies**		
Nulligravid	28	6.4
1-3	264	61.0
≥4	141	32.6
**Number of deliveries (n= 433)**		
Nulliparous	54	12.5
1-3	284	65.6
≥4	95	21.9
**Abortion (n= 432)**	167	38.7
**Time of HIV diagnosis ≥ 5 years (n=435)**	228	52.4
**CD4+ T-lymphocyte ≥ 350 (cells/μl) (n= 401)**	309	77.1
**HIV viral load < 1000 copies/ml (n= 403)**	309	76.7
**Antiretroviral use (n= 433)**	414	95.6

^a^HIV, human immunodeficiency virus. ^b^Benefit salary: illness aid, unemployed benefit, retired. ^c^The number of women in each category may not add up to 435 due to missing information.

Considering only active toxoplasmosis (IgM); rubella (IgM); hepatitis B, hepatitis C, and syphilis infections; and TB cases from SINAN-Sergipe, 85 (19.5%) of the 435 had cases of coinfections. Eighty (94.1%) of the 85 patients had one type of coinfection, and 5 (5.9%) had two or more types.

The prevalence rates were as follows: syphilis (38/9.1%), TB (17/3.9%), toxoplasmosis (13/3.8%), hepatitis C (10/2.5%), hepatitis B (9/2.3%), and rubella (5/1.8%). Additionally, we identified the seropositivity for the IgG antibody of cytomegalovirus (300/96.2%), rubella (252/90.0%), and toxoplasmosis (242/71.2%). When associating the type of coinfection with the time of HIV diagnosis, a statistically significant effect was observed for TB and hepatitis C coinfections. The proportion of HIV-positive women who were coinfected and those who were not coinfected with TB and hepatitis C differed according to the time of HIV diagnosis (Table 2).

**TABLE 2:** Prevalence of coinfections and association with the time of HIV^a^ diagnosis, Sergipe, Brazil, August 2014-November 2017.

			Time of HIV diagnosis				
	Prevalence		< 5 years		≥ 5 years		
Coinfections	N^f^	%	N^f^	%	N^f^	%	P-value^d^
**Cytomegalovirus (IgG** ^b^ **) (n= 312)**							
Reagent	300	96.6	182	60.7	118	39.3	0.233^e^
Non-reagent			5	41.7	7	58.3	
**Rubella (IgG) (n=280)**							
Reagent	252	90.0	170	67.5	82	32.5	0.065
Non-reagent			14	50.0	14	50.0	
**Rubella (IgM** ^c^ **) (n=280)**							
Reagent	5	1,8	3	60.0	2	40.0	1.0^e^
Non-reagent			181	65.8	94	34.3	
**Toxoplasmosis (IgG) (n=340)**							
Reagent	242	71.2	148	61.2	94	38.8	0.736
Non-reagent			58	59.2	40	40.8	
**Toxoplasmosis (IgM) (n=340)**							
Reagent	13	3.8	9	69.2	4	30.8	0.516
Non-reagent			197	60.2	130	39.8	
**Syphilis (n=419)**							
Yes	38	9.1	22	57.9	16	42.1	0.330
No			189	49.6	192	50.4	
**Tuberculosis (n=435)**							
Yes	17	3.9	4	23.5	13	76.5	0.025
No			214	51.2	204	48.8	
**Hepatitis B (n=394)**							
Reagent	9	2.3	5	55.6	4	44.4	1.0^e^
Non-reagent			202	52.5	183	47.5	
**Hepatitis C (n=401)**							
Reagent	10	2.5	1	10.0	9	90.0	0.008^e^
Non-reagent			209	53.5	182	46.5	

^a^ **HIV:** human immunodeficiency virus. ^b^ **IgG:** immunoglobulin G. ^c^ **IgM:** immunoglobulin M. ^d^Pearson’s chi-squared test. ^e^Fisher’s exact test. ^f^The number of women in each category may not add up to 435 due to missing information.

When comparing the data of the HIV-positive women who had at least one coinfection with those who had no coinfections, it was observed that women who self-reported to be black (PR, 1.58; 95% CI, 0.75-3.33), those exposed to HIV through sexual intercourse (PR=1.58; 95% CI, 0.80-2.73), those who had their first sexual intercourse when they were younger than 15 years old (PR=1.51; 95% CI, 0.93-2.45), or those who had sex for money (PR=1.76; 95% CI, 0.88-3.50) were more likely to have a coinfection (Table 3).

**TABLE 3:** Socioeconomic, clinical, and risk behavior factors associated with the presence or absence of coinfection in women living with HIV^a^, Sergipe, Brazil, August 2014-November 2017.

	Coinfection^b^					
	Presence		Absence			
Variables	N^e^	%	N^e^	%	PR^c^	95% CI^d^
**Race/ethnicity (n= 393)**						
Black	34	28.4	86	71.6	1.58	0.75-3.33
Mixed	33	15.5	180	84.5	0.73	0.35-1.57
White	12	20.0	48	80.0	1	-
**Schooling (n=431)**						
≤ 8 years	76	20.0	304	80.0	1.17	0.54-2.50
> 8 years	9	17.6	42	82.4	1	-
**Living place (n=431)**						
Rural	13	17.8	60	82.2	1.16	0.60-2.23
Urban	72	20.1	286	79.9	1	-
**Inadequate housing (n=435)**						
Yes	14	19.7	57	80.3	1.01	0.53-1.92
No	71	19.5	293	80.5	1	-
**Income (n=432)**						
No income	6	18.2	27	81.8	0.92	0.36-2.29
With income	78	19.5	321	80.5	1	-
**HIV exposure category (n=411)**						
Sexual intercourse	69	20.9	260	79.1	1.48	0.80-2.73
Vertical transmission	1	14.3	6	85.7	0.93	0.10-8.31
Unknown	15	15.2	84	84.8	1	-
**Age at first sexual intercourse (years old) (n=421)**						
≤ 15	44	23.0	147	77.0	1.51	0.93-2.45
> 15	38	16.5	192	83.5	1	-
**Sex for money (n=430)**						
Yes	13	28.9	32	71.1	1.76	0.88-3.50
No	72	18.7	313	81.3	1	-
**Drug use (n=433)**						
Yes	16	23.2	53	76.8	1.33	0.71-2.49
No	57	18.5	251	81.5	1	-
**Time of HIV diagnosis (years) (n=435)**						
< 5	40	18.4	178	81.6	1	-
≥ 5	45	20.7	172	79.3	1.16	0.72-1.87

^a^HIV, human immunodeficiency virus. ^b^Positive results were considered for toxoplasmosis (IgM), rubella (IgM), hepatitis B, hepatitis C, and syphilis in addition to reported cases of tuberculosis by SINAN. ^c^PR, prevalence ratio. ^d^CI, confidence interval. ^e^The number of women in each category may not add up to 435 due to missing information.

In Sergipe, one-fifth of the participants had some types of coinfection. In individuals living with HIV/AIDS, coinfection was an expected phenomenon because HIV infection deteriorates the immune system. However, this condition can be minimized with the timely and immediate use of ART. Studies have shown a decrease in opportunistic infections and a reduction in mortality in HIV-infected individuals after the advent of ART^1^. Despite the relatively insufficient studies regarding the magnitude of coinfections among the female population, the findings in a US cohort of HIV-positive women and their children were consistent with the previously reported finding^5^. In this context, despite advancements in the treatment of people living with HIV, coinfections are still common, leading to deaths^2^. The prevalence of coinfections can vary according to the etiological agent, even within the same population. In this study, most coinfections were prevalent between 1.8 and 3.9, except for syphilis, which had a rate of 9.1. A high prevalence of syphilis in women living with HIV was also reported by a study conducted in the Amazon region of Brazil^6^.

Based on our findings, some interpretations can be offered. A possible explanation for the high prevalence of syphilis in women living with HIV in this study is due to the number of syphilis cases in the Brazilian population that have increased in recent years. Additionally, both HIV and syphilis diseases share the same risk factors, suggesting a higher likelihood of coinfection occurring, emphasizing the importance of tracking this coinfection in this subpopulation.

It is well established that risky behaviors such as needle sharing, abuse of psychoactive substances (alcohol and drugs), multiple partners, prostitution, and poor adherence to condom use increase the chances of acquiring sexually transmitted infections. In this study, more than half of the women reported not using condoms during all sexual encounters. Studies show that not using condoms is mainly due to the belief that it is unnecessary to use condoms among heterosexual HIV-seroconcordant couples and due to gender inequality as often the women are in long-term and oppressive relationships, where negotiating condom use with the partner can be difficult^7^.

Despite not being the most prevalent coinfection in this study, TB remains an important health problem. A national population-based study^8^ using probabilistic linkage technique found an estimated 6.3% of TB prevalence in women living with HIV between 2011 and 2014, which was higher than the findings of this study. This suggests that the prevalence of TB/HIV coinfection varies widely among Brazilian regions.

In Sergipe, the relatively high prevalence of TB in women living with HIV may be the result of the adherence to Brazilian public policies on HIV and TB control, which include timely use of ART, intensive screening for latent TB infection, and early diagnosis and immediate treatment of TB with chemoprophylaxis, preventing the development of active TB infection. In fact, Sergipe is considered as one of the Brazilian states with the highest rates of ART use in coinfected individuals during the treatment of TB^9^.

The effect of ARV on the survival of individuals living with HIV also confirmed the development of chronic hepatitis caused by viruses B and C in this population, specifically in developing regions and in regions where there is a high endemicity of HIV infection. In African areas, studies show that the overall prevalence of HBsAg in adult women can range from 7% to 14%^10^ ^,^ ^11^. A Brazilian national study found that women infected with HIV were less likely coinfected with hepatitis B or C^12^.

Other findings worth noting were the high frequencies of seropositivity for cytomegalovirus, rubella, and toxoplasmosis IgG antibodies. This is very relevant, specifically for cytomegalovirus and toxoplasmosis because there is a risk of latent reactivation for these infections^13^ ^,^ ^14^. However, the high seropositivity for the rubella IgG antibody may correspond to the immunity acquired through vaccination or prior infection, which may explain the low prevalence of active infection in our study.

There have been few published studies regarding active rubella-HIV coinfection; however, several studies have analyzed the seroprevalence of the IgG antibody to evaluate the response to the rubella vaccine in individuals living with HIV, which showed similar and consistent results with our study for rubella IgG positivity. One of them identified that 89.3% were seropositive for rubella IgG and 10.69% were susceptible to coinfection^15^.

It is clear that it is not only important to understand the epidemiological characteristics of HIV-positive women but also be aware of the proportion of coinfections in this population group. However, this study has some limitations. Although we conducted face-to-face interviews, clinical information was completed from medical records and surveillance databases. Second, it was difficult to find the records of all the test results for coinfections. Conversely, this missing information occurred randomly; hence, the principal consequence was loss of power. In this sense, it was assumed that these tests may not have been performed or their results were not included in the medical records, which may characterize failures in the coinfection screening protocol. Third, this study may not have been able to determine the true magnitude of the prevalence of these coinfections in the entire female population living with HIV in the Sergipe State because most of the women evaluated were routinely followed up and reported to be using ART. However, the final sample corresponded to more than half of the women enrolled in the service and was almost double the calculated sample size. Despite these limitations, this study determined the prevalence rates and important characteristics that can guide the care provided and the planning of activities for women living with HIV.

In conclusion, in Sergipe, one-fifth of the women living with HIV had some types of coinfection, with prevalence ranging from 1.8 to 3.9, except for syphilis, which had the highest rate of 9.1. Additionally, high seroprevalence for IgG antibody for cytomegalovirus, rubella, and toxoplasmosis was identified. Our results provide potential strategies to improve control programs for HIV by targeting interventions to population, with strengthening of public policies for the prevention, control, diagnosis, and treatment of coinfections in HIV-positive women with different characteristics.
